# *PLCζ, WBP2NL* and *TNF-α* expression in spermatozoa is associated with stallion fertility and seminal quality?

**DOI:** 10.1590/1984-3143-AR2023-0088

**Published:** 2024-04-12

**Authors:** Verônica La Cruz Bueno, Henrique Boll de Araujo Bastos, Luiz Augusto Centeno, Nélson Alexandre Kretzmann, Rodrigo Costa Mattos, Sandra Fiala Rechsteiner

**Affiliations:** 1 Histologia e Reprodução Equina, Departamento de Morfologia, Instituto de Biologia, Universidade Federal de Pelotas, Pelotas, RS, Brasil; 2 Laboratório de Reprodução Animal, Universidade Federal do Rio Grande do Sul, Porto Alegre, RS, Brasil

**Keywords:** stallions, gene expression, conception rate, seminal quality

## Abstract

This study aims to investigate the gene expression of sperm-borne *phospholipase C zeta* (*PLCζ*), WW domain-binding protein 2N-Terminal Like (*WBP2NL*), and Tumor necrosis factor (*TNF-α*), as a negative control, in spermatozoa and their relationship with fertility and seminal quality in stallions. Ejaculates from 40 Criollo stallions were used, whose fertility was assessed on the basis of their pregnancy rate per cycle in at least two breeding seasons. Pregnancy rates ranged from 20% to 90% and were used to divide the stallions into two groups: High rates (≥ 50%) (n = 25), and Low rates (< 50%) (n = 15). A computer-assisted sperm analysis system - (CASA) analyzed semen after collection. Also were evaluated the physical and functional integrity of the plasmatic membrane and sperm morphology alterations. All stallions expressed *PLCζ, WBP2NL,* and *TNF-α*. *PLCζ* positively correlates with conception rate, total motility (TM), progressive motility (PM), plasmatic membrane functionality, and integrity. A simple linear regression was detected between pregnancy rate and *PLCζ* expression (P = 0.003), TM (P < 0.001) and PM (P < 0.001). *PLCζ* gene expression was higher (P = 0,012) in the High rates group than in the Low group. *WBP2NL* and *TNF-α* did not correlate with seminal quality and stallion’s fertility. It was concluded that *PLCζ* gene expression in the spermatozoa might be used as a biomarker of fertility and seminal quality in stallions. Parameters of sperm kinetics also showed, positive correlation between TM, PM and pregnancy rate.

## Introduction

Stallions are selected as sires based on three qualities: pedigree, performance record, and conformation (Varner et al., 2008); however, fertility or fertility potential is usually, at best, secondary considerations (Colenbrander et al., 2003). Fertility can be determined by parameters that reflect breeding success rates (Hamann et al., 2005) or by using sperm characteristics, which have reported inconsistent results (Suliman et al., 2018).

In the last decades, there was progress in assisted reproduction techniques for the production of foals (Hinrichs, 2012). Global transcriptome in spermatozoa from fertile stallions was described in the last years, and its essential role in male fertility explored (Das et al., 2013).

In all mammalian species studied, fertilization results in a series of intracellular calcium (Ca^2+)^ increases, referred to as oscillations, triggered by *PLCζ*, responsible for driving oocyte activation (Bedford-Guaus et al., 2011). The oscillation of Ca^2+^ levels plays an essential role in early embryonic development (Gat and Orvieto, 2017). Lower RNA expression of *PLCζ* and protein levels was observed in teratozoospermic infertile men when compared with fertile patients (Azad et al., 2018; Kamali-Dolat et al., 2016); however, no differences were detected in localization patterns (Azad et al., 2018). A decrease of *PLCζ* expression was demonstrated in a single stallion with reduced conception rates in field trial comparing with a unique control (Gradil et al., 2006).

Gene *WBP2NL*, which encodes PAWP/WBP2NL (a sperm-borne tryptophan domain-binding protein), is related to fertilization. This gene was also singled out as a possible trigger to Ca2+ oscillations during oocyte activation in the fecundation process (Wu et al., 2007; Aarabi et al., 2014). A strong positive correlation was found between PAWP/WBP2NL abundance levels and fertilization rates after ICSI in humans (Aarabi et al., 2014), with high levels of PAWP/ WBP2NL associated with higher fertilization rates (Tavalaee and Nasr-Esfahani, 2016). However, to our knowledge, the expression of *WBP2NL* has not been described in the equine spermatozoa.

Tumor necrosis factor (TNF-α) is a cytokine with essential roles in homeostasis and disease pathogenesis, being one of the most-documented cytokines in spermatogenesis (Białas et al., 2009; Bami et al., 2017). TNF-α is also present in canine spermatozoa (Payan-Carreira et al., 2012) and increasing concentrations in seminal fluid has been reported to reduce progressive motility on human spermatozoa (Lampião and du Plessis, 2008; Seshadri et al., 2009). This study investigates the gene expression of *PLCζ, WBP2NL,* and *TNF-α*, as a negative control, in spermatozoa and their relationship with fertility and seminal quality, in stallions.

## Materials and methods

### Animals

A total of 40 Criollo stallions were used, with a mean age from 9.1 ± 1.0 years old (4 to 18 years old) and weighing 450 to 500 Kg. Feeding was done daily with concentrate and alfalfa hay, with animals also having free access to water and mineral supplementation. The animals were housed in breeding centers in Rio Grande do Sul (30ºS, 51ºW), Brazil. The Committee of Ethical Use in Animal Experimentation at Universidade Federal de Pelotas, Rio Grande do Sul, Brazil approves the study (protocol number 2753).

### Experimental design

Fertility evaluation of selected stallions happened through their reproductive history, based on pregnancy rate per cycle on the 16^th^ day after artificial insemination in at least 30 inseminated mares per stallion (Table 1). Were considered at least two breeding seasons. Pregnancy rates ranged from 20% to 95% and were used to divide the stallions into two groups: High rates, the pregnancy rate per cycle ≥ 50% (n = 25), and Low rates, with pregnancy rate per cycle 50% (n = 15).

**Table 1:** Details of the sequences used for quantitative real-time polymerase chain reaction amplification of mRNA from stallion’s sperm cells.

**Gene**	**Sequences**	**Temp**	**References**
***PLCζ***	F:5’-AAGGATGCCGTTGTCTGGAA-3’	57°C	Genbank (2018a)
	R:5’-CCGGGTAGTCAGAGGTAATGA-3’		
***WBP2NL***	F:5’-CTCAGTCAACGATCCCATGCT-3’	60°C	Genbank (2018b)
	R:5’-GTCCTTCCCAGCCACCATCTG-3’		
***TNF-α***	F: 5’-GCTCCAGACGGTGCTTGTG-3’	57.5°C	Riihimäki et al. (2008)
	R: 5’-GCCGATCACCCCAAAGTG-3’		

F: f*orward* primer; R: r*everse* primer; Temp: annealing temperature.

One ejaculate was collected from each stallion in reproductive activity during the second breeding season after two days of sexual rest. Semen was collected with an artificial vagina. Immediately after collection, the ejaculate was filtered, placed in a Falcon® tube, and forwarded to the laboratory. For fresh semen analysis was used 1 mL of each ejaculate.

The remainder ejaculate was used, for gene expression quantification by qPCR. It was transferred to Falcon® tubes (15 mL) and centrifuged at 400 x g for 10 min. The resulting supernatant was discarded, the pellet on the Falcon tube resuspended in PBS and centrifuged once again. This procedure was repeated three times. The remaining pellet from the centrifugations was then resuspended in 2 mL RNAlater® (Life Technologies) in RNase-free cryotubes and stored at -80ºC for later analysis. At least 750 x 10^6^ spermatozoa from each stallion were stored.

### Semen analysis

Sperm concentration was determined using a hemocytometer chamber. Total Motility (%) (TM); Progressive Motility (%) (PM); Fast Motility (%) (FM); Slow Motility (%) (SM); Local Motility (%) (LM); Average Path Velocity (VAP, μm/s); Straight Line Velocity (VSL, μm/s); Curvilinear Velocity (VCL, μm/s); Amplitude of Lateral Head Displacement (ALH, μm); Beat Cross Frequency (BCF, Hz); Straightness (STR, %) (VSL/VAP); Linearity (LIN, %) (VSL/VCL); Fast Velocity (VAPE, %) were evaluated via CASA system (Computer Assisted Sperm Analysis, Tiefenbach, Germany, AndroVision®, Minitube).

The physical integrity of the plasmatic membrane was analyzed by incubating 400 μL of semen with 3 μL of propidium iodide (PI) and 2 μL of carboxyfluorescein diacetate (CFDA) at 37°C for eight min. Epifluorescence microscopy (1000x) evaluated 100 spermatozoa per sample. Were considered intact sperm, cells with green coloring, and red-stained as damaged cells (Garner et al., 1986).

The functional integrity of the plasmatic membrane was assessed using a hypoosmotic-swelling test (HOST). Distilled water (200 μL) were added to 100 μL of semen (osmolarity: 100 mOsmol kg -1) and incubated at 37ºC for 8 min. Samples were analyzed in a phase-contrast microscope (400x). Were evaluated 100 spermatozoa per sample, and cells with coiled tails were considered intact (Lagares et al., 1998).

Sperm morphology was evaluated with Diff-Quick stain (Laborclin, Brazil): slides with smear samples of semen were immersed in the stain for 10 sec and being immediately analyzed with an optical microscope with an immersion objective (1000x). One hundred sperm cells were counted from each sample. Sperm defects were classified as either major or minor in terms of their perceived adverse effects upon male fertility (Blom, 1973).

### qPCR

The mRNA of the 40 stored samples was extracted via commercial kit Trizol® (Thermo Fisher Scientific) according to the manufacturer's instructions. The concentration of total RNA extracted was quantified via spectrophotometry using a NanoVue® (GE Healthcare Life Sciences). Only RNA samples with a 260/280 ratio between 1.9 and 2.1 were used to minimize the adverse effects of protein contamination.

Conversion to cDNA was made via commercial kit SuperScript III Reverse Transcriptase® (Thermo Fisher Scientific), according to the manufacturer’s instructions. qPCR technique analyzed gene expressions of *PLCζ, WBP2NL,* and *TNF-α* in the sperm cell . Amplification was performed via Real-Time PCR Applied Biosystems Thermal Cycler® (Thermo Fisher Scientific), with data processed by the integrated database v2.3. Lastly, cDNA amplification was conducted via a primer specifically designed for an amplicon (sequence of interest) using fluorophore SYBRTM Green Master Mix (Thermo Fisher Scientific). Primers were obtained via Integrated DNA Technologies (IDT®), and sequences employed are listed in Table 1. The RNA sequence used to design primers was verified through blast suit: PREDICTED (Genbank, 2018a) *Equus caballus 1-phosphatidylinositol 4,5-bisphosphate phosphodiesterase zeta-1-like* (LOC111767502), ENSECAG00000011373 transcript variant X6, mRNA Sequence ID: XM_023643199.1; PREDICTED (Genbank, 2018b): *Equus caballus WBP2 N-terminal like*, transcript variant X2, mRNA Sequence ID: XM_023631051.1, all with 100% efficiency.

The program used for amplification was 95ºC per 2 min, followed by 40 cycles of denaturation at 95ºC per 15 sec, annealing per 30 sec, and extension at 60ºC per 30 sec. Absolute quantitative qPCR results were determined using the standard curve formula=10^((ct target CT standard)/slope) (Bastos et al., 2014).

### Statistical analysis

Prism 8.3.0 (Graph Pad Software Inc. San Diego, CA, USA) performed the statistical analysis. Pearson´s coefficient analyzed correlations between *PLCζ, WBP2NL, TNF-α* expression, and pregnancy rates, seminal parameters (TM, PM, FM, SM; LM; VAP; VSL; VCL; ALH; BCF; STR; LIN; VAPE), plasmatic membrane integrity and functionality, major and minor defects. Fischer exact test was used to compare *PLCζ* expression > 200 and < 200 in stallions of both groups (High and Low). A simple linear regression was performed between pregnancy rates and *PLCζ* expression, TM and PM. A t-test was calculated between High and Low pregnancy rates groups and each gene (*PLCζ, WBP2NL,* and *TNF-α*) expression. Results were expressed as mean ± SEM; *P* ˂ .05 was regarded as significant.

## Results

Table 2 show clinical spermatic parameters of stallions from the High and Low rates groups. In stallions of the High rate group PR, TM, PM, SM, CFDA, and HOST were higher (P < 0.047) than in the Low rates group.

**Table 2:** Mean pregnancy rate and clinical spermatic parameters of stallions classified as High and Low rates group.

**Parameters**	**Groups**		**Probability**
	**High (n = 25)**	**Low (n = 15)**	
**PR**	65.7 ± 2.6	36.3 ± 1.8	< 0.001
**TM**	72.2 ± 2.8	43.3 ± 3.7	< 0.001
**PM**	50.9 ± 3.8	27.3 ± 3.2	< 0.001
**SM**	31.0 ± 2.6	20.8 ± 2.3	= 0.047
**CFDA**	60.4 ± 2.5	34.9 ± 3.4	< 0.001
**HOST**	59.6 ± 3.2	33.4 ± 2.9	< 0.001

PR - pregnancy rate; TM - total motility; PM - progressive motility; SM - slow motility; CFDA – plasma membrane physical integrity; HOST - plasma membrane functional integrity.

All stallions expressed *PLCζ*. Pearson’s correlation coefficient (R) and Probability (P) for *PLCζ* gene expression levels in sperm cells of Criollo stallions and pregnancy rate and seminal parameters are depicted in Table 3. Pregnancy rate was positively correlated with *PLCζ* expression in sperm cells (R = 0.454; P = 0.007). Likewise, total motility (R = 0.386; P = 0.014), progressive motility (R = 0.413; P = 0.008), slow motility (R = 0.427; P = 0.006), physical integrity (R = 0.384; P = 0.014) and functional integrity (R = 0.449; P = 0.004) were positively correlated with *PLCζ* expression. No correlation was observed (P > 0.05) between *PLCζ* expression and FM; LM; VAP; VSL; VCL; ALH; BCF; STR; LIN; VAPE, major and minor defects.

**Table 3:** Pearson’s correlation coefficient (R) and Probability (P) for *PLCζ* gene expression levels in sperm cells and pregnancy rate and seminal parameters.

	**PR**	**TM**	**PM**	**SM**	**CFDA**	**HOST**
**R**	0.454	0.386	0.413	0.427	0.384	0.449
**P**	0.007	0.014	0.008	0.006	0.014	0.004

PR - pregnancy rate; TM - total motility; PM - progressive motility; SM - slow motility; CFDA – plasma membrane physical integrity; HOST - plasma membrane functional integrity.

**Figure 1:**
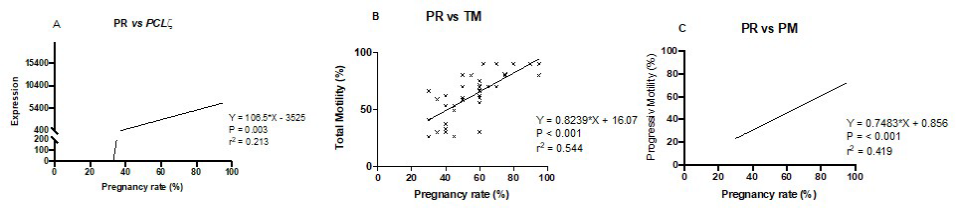
Scatter plot for pregnancy rate (PR) and its relationship with: (A) gene expression of *PLCζ* in sperm cells of stallions; (B) Total Motility (TM) and (C) Progressive motility (PM).

**Figure 2:**
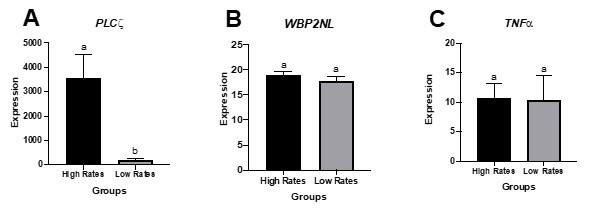
Gene expression of *PLCζ* (A), *WBP2NL* (B), *TNF-α* (C) in the sperm cells of stallions from the High rates group (n= 25) and from the Low rates group (n= 15). (a, b) (P = 0.012) different letters in each gene represent a significant difference between groups.

Figure 1A depicts a scatter plot for *PLCζ* gene expression and its relationship with pregnancy rates . Stallions with the highest expression of *PLCζ* presented the highest pregnancy rate. A significant (P = 0.003) linear regression was observed with the following equation Y = 106.5*X - 3525 (R^2^ = 0.213). Figure 2B and 2C shown linear regressions (P < 0.001) of pregnancy rate and TM (Y = 0.8239*X + 16.07; R^2^ = 0.544) and pregnancy rate and PM (Y = 0.7483*X + 0.856; R^2^ = 0.419).

In the High rates group, stallions presenting *PLCζ* gene expression > 200 (n = 20; 86.9%) differ (P = 0.001) from stallions with lower *PLCζ* expression (n = 5; 29.4%), (Table 4). Of the 25 stallions with High pregnancy rates/cycle, 20 (80%) presented high *PLCζ* expression, and 5 (20%) presented low expression of the gene. Fifteen stallions were classified in the Low PR group; 12 (80%) presented low expression of the gene, and 3 (20%) presented high expression. Stallions of the High rates group presented higher (P = 0.012) *PLCζ* expression than stallions of the Low rates group (Figure 2A).

**Table 4:** Distribution of the stallions of the High rates group (≥ 50% pregnancy rate) and the Low rates group (≤ 45% pregnancy rate) with the expression of the *PLCζ* gene in the spermatozoa.

**Gene Expression**	**Stallions (n)**	**High rates group (≥ 50% PR)**		**Low rates group (< 50% PR)**	
		**n**	**%**	**n**	**%**
**˃ 200**	23	20	86.9^a^	3	13.1^a^
**˂ 200**	17	5	29.4^b^	12	70.6^b^

(a, b) (P < 0.001) different letters represent significant difference. PR – Pregnancy rate.

No correlation was observed between *WBP2NL* and *TNF-α* with pregnancy rate and seminal parameters. No differences (P = 0.319) were observed in *WBP2NL* and *TNF-α* expression, within the High and the Low rates groups (Figure 2B and 2C).

## Discussion

The present study demonstrated the gene expression of *PLCζ*, *WPB2NL, and TNF-α* in the equine spermatozoa. The fertility of each stallion was recorded from at least 30 mares, which added up to more than 1,200 inseminated mares.

Poor fertility of breeding stallions is a recognized problem in the equine industry. Among farm animals, horses have the lowest reproductive rate (Suliman et al., 2018). For a long time, the male genome was believed to be the only material introduced into the oocyte cytoplasm that held a determining role in the fecundation process. The discovery of the fact that spermatozoa also introduces centrioles (Simerly et al., 1995), soluble factors that activate the oocyte (Saunders et al., 2002), and RNAs (Ostermeier et al., 2004), changed that perception.

At fertilization, sperm is responsible for triggering a series of calcium ions increases, referred to as oscillations (Bedford-Guaus et al., 2012). These oscillations are responsible for oocyte activation and the initiation the embryonic development (Gradil et al., 2006). Sperm delivered protein responsible for these events in mammals is PLC*ζ*Bedford-Guaus et al. (2012). A higher expression of *PLCζ* gene, in sperm cells of stallions, in the High rates group, was observed in the present study. All stallions expressed *PLCζ*; the lower expression correlates with low fertility.

A survey was conduted genome-wide association study for estimated breeding values of the paternal component of the pregnancy rate per estrus cycle (EBV-PAT) in Hanoverian stallions. A total of 228 Hanoverian stallions were genotyped using the Equine SNP50 Beadchip. The most significant association was found on horse chromosome 6 for a single nucleotide polymorphism (SNP) within phospholipase C zeta 1 (PLCz1). In the close neighbourhood to PLCz1 is located CAPZA3 (capping protein (actin filament) muscle Z-line, alpha 3). The gene PLCz1 encodes a protein essential for spermatogenesis and oocyte activation through sperm induced Ca2+ -oscillation during fertilization. The noncoding polymorphisms within PLCz1 were identified as conferring stallion fertility and PLCz1 as candidate locus for male fertility in Hanoverian warmblood. (Schrimpf et al., 2014).

*PLCζ* was already observed in the soluble fractions of sperm from mice (Saunders et al., 2002), humans (Heindryck et al., 2012) pigs (Kaewmala et al., 2012), cats (Villaverde, 2010), and horses (Bedford-Guaus et al., 2011). In equine species, this gene is expressed over the acrosome, equatorial segment, head-midpiece junction, and the principal piece of the flagellum (Bedford-Guaus et al., 2011). In horses, intracytoplasmic sperm injection (ICSI) is the elective method for assisted fertilization. Sperm *PLCζ* amount exhibits a large variation from stallion to stallion which can influence fertility outcomes. Stallion sperm samples showing low *PLCζ* consistently result in lower fertility after ICSI (Gonzalez-Castro and Carnevale, 2023).

*PLCζ* expressed over the flagellum is catalytically active and may be unique to the horse. Based on this observation, it was hypothesized that *PLCζ* might play a role in processes other than oocyte activation, such as hyperactivation (Bedford-Guaus et al., 2012). Hyperactivation is a possible explanation to the positive correlation of *PLCζ* expression with TM; *PLCζ* expression correlates with sperm motility in men (Heindryck et al., 2012). No correlation was observed between *PLCζ* expression and velocity parameters (VAP, VSL, VCL) and ALH; these results agree with the observed previously (Barrier Battut et al., 2016), which detected no correlation between the same parameters and fertility.

*PLCζ* gene expression exhibited a correlation with the functional and physical integrity of the plasmatic membrane. The integrity of the sperm membrane is crucial for the maintenance of sperm fertilizing capability (Barrier Battut et al., 2016) and the preservation of cellular homeostasis. In this way, plasmatic membrane integrity exerts a vital role on sperm survival in the female reproductive tract, and on the preservation of its fertility capability (Celeghini et al., 2010). Sperm functional and morphological integrity evaluation is useful for equine semen routine analysis and could aid in predicting semen fertility (Lagares et al., 2000). However, pregnancy rate predictability is higher when used TM and PM than *PLCζ.*

Stallion sperm cells from the High and Low rates groups expressed the *WBP2NL* gene. Its expression was not correlated with conception rate and semen quality, agreeing with studies in humans that described the lack of correlation between this gene expression and fertility (Freour et al., 2017), and with studies in mouse where no correlation was observed between the expression of this gene and semen quality. This result disagrees with two studies in humans that observed a correlation between fertility and the presence of the protein PAWP/WBP2NL, coded by the gene *WBP2NL* (Aarabi et al., 2014; Tavalaee and Nasr-Esfahani, 2016) The lack of correlation between *WBP2NL* gene and conception rates may be explained by the absence of Ca2+ oscillations, once this gene is not capable of initiating these oscillations to activate the oocyte (Satouh et al., 2015; Escoffier et al., 2016).

*TNF-α* has no relationship with calcium oscilation and was used in this experiment as a negative control. The present study showed that all stallions express *TNF-α*, suggesting that this expression is important to spermatic physiology. However, a correlation between *TNF-α* gene expression and seminal quality and fertility in stallions was not observed. This absence may be explained by the category of stallions used, since cytokines are related to inflammatory processes, and all the stallions used in this experiment were healthy, with no clinical alterations. Studies have shown that *TNF-α* is detrimental for progressive sperm motility (Lampião and du Plessis, 2008). *TNF-α* affects spermatogenesis by changing the structure of the blood-testis barrier and apical ectoplasmic specialization of Sertoli cells, which may lead to abnormal spermatogenesis (Li et al., 2006).

Mature mammalian spermatozoa contain a complex population of RNAs, some of which might regulate spermatogenesis, while others probably play a role in fertilization and early embryonic development (Das et al., 2013). Spermatozoa are loaded with RNAs, but in most cases, their roles are unknown (Hosken and Hodgson, 2014). Their nucleus contains diverse RNA populations (Dadoune, 2009): messenger RNA, microRNA (miRNA), interference RNA, and antisense RNA (Hosken and Hodgson, 2014), that have been transcribed throughout spermatogenesis and are delivered by the sperm to the oocyte at fertilization (Dadoune, 2009). However, the miRNA of the spermatozoa modified as the cells descends through the epididymis, including the apparent loss and acquisition of miRNA between the proximal and distal epidydimal segments (Nixon et al., 2015). The exposure of various forms of stress during the extend residence in epidydimal environment may be capable to alter sperm miRNA content (Nixon et al., 2015).

Studying genes of importance for spermatogenesis may help us gain insight regarding the underlying molecular cause of certain unexplained cases of male subfertility and infertility (Petit et al., 2015). With this in mind, we may be able to develop biomarkers that can help to identify these individuals (Suliman et al., 2018) enabling more specific management and proper selection.

In summary, *PLCζ, WBP2NL*, and *TNF-α* were expressed in spermatozoa of all stallions. *PLCζ* exhibited a correlation with pregnancy rate, motility parameters, and plasma membrane integrity and functionality, presenting higher expression in stallions with a high pregnancy rate. *WBP2NL* and *TNF-α* did not correlate with any of the parameters evaluated. It was concluded that *PLCζ* gene expression in equine spermatozoa might be utilized as a biomarker for seminal quality and fertility in stallions. Parameters of sperm kinetics also showed, positive correlation between TM, PM and pregnancy rate.
